# The sex-specific role of adrenal androgens in youth psychopathology

**DOI:** 10.1038/s41398-026-04121-3

**Published:** 2026-06-04

**Authors:** Franka Edith Weisner, Bianca Serio, Sofie Valk, Luise Bläschke, Franziska Degenhardt, Anke Hinney, Raphael Hirtz, Lars Dinkelbach

**Affiliations:** 1 Institute of Sex- and Gender-sensitive Medicine, University Hospital Essen, University of Duisburg-Essen, Essen, Germany; 2 Max Planck Institute for Human Cognitive and Brain Sciences, Leipzig, Germany; 3 Max Planck School of Cognition, Leipzig, Germany; 4 Institute of Neuroscience and Medicine, Brain & Behavior (INM-7), Research Centre Jülich, Jülich, Germany; 5 Institute of Systems Neuroscience, Medical Faculty, Heinrich Heine University Düsseldorf, Düsseldorf, Germany; 6 Department of Child and Adolescent Psychiatry, Psychosomatics and Psychotherapy, LVR University Clinic Essen, University of Duisburg-Essen, Essen, Germany; 7 Section of Molecular Genetics in Mental Disorders, University Hospital Essen, University of Duisburg-Essen, Essen, Germany; 8 Center for Translational Neuro- and Behavioral Sciences, University Hospital Essen, University of Duisburg-Essen, Essen, Germany; 9 Center for Child and Adolescent Medicine, Helios University Hospital Wuppertal, Witten/Herdecke University, Wuppertal, Germany; 10 Department of Pediatrics II, University Hospital Essen, University of Duisburg-Essen, Essen, Germany

**Keywords:** Predictive markers, Psychiatric disorders, Molecular neuroscience

## Abstract

Adolescence is a vulnerable period for the emergence of mental health problems. Adrenarche, an early stage of pubertal development marked by rising adrenal androgens, particularly dehydroepiandrosterone (DHEA), may influence emotional and behavioral development. However, longitudinal evidence linking early-adolescent endocrine influences to adolescent psychopathology remains limited. Using data from the Adolescent Brain Cognitive Development (ABCD) Study (up to *N* = 11 696), we analyzed whether salivary DHEA during early adolescence predicted later externalizing and internalizing symptoms during adolescence. Early-adolescent hormone levels were averaged across baseline and 1-year follow-up (age range = 8.9–12.4 years). Outcomes were measured via the Child Behavior Checklist (CBCL) at the 2-, 3-, and 4-year follow-ups (up to = 14.08 ± 0.68 years). Sex-stratified linear mixed models adjusted for age, race/ethnicity, BMI and physical activity. In males, higher DHEA levels were linked to fewer externalizing symptoms across follow-ups (e.g., β = –0.07 SD change of CBCL per SD-change of log-transformed DHEA levels (95% CI [–0.10, –0.04] at 3-year) and fewer internalizing symptoms at 3-year and 4-year follow-ups. Higher early-adolescent DHEA in males also reduced the probability of externalizing symptoms to reach clinical thresholds across follow-ups (e.g., adjusted Risk Ratio = 0.81 to reach clinical threshold for CBCL externalizing per SD increase in log-transformed DHEA; 95% CI [0.70, 0.93] at 3-year). In females, no hormone–symptom associations emerged. Sex-by-DHEA interaction effects tended to increase across follow-up years for both symptom domains. These findings suggest that early-adolescent adrenal endocrine influences may contribute to the development of sex-specific vulnerability during adolescence. Future studies should consider adrenarche as a sensitive period for hormonal effects on mental health.

## Background

Adolescence marks a critical developmental window during which many mental health problems begin to emerge [1, 2]. This period is also characterized by a notable shift in mental health vulnerability between the sexes [3–6]. During childhood, boys typically exhibit higher levels of psychiatric symptoms, especially externalizing behaviors such as aggression and hyperactivity [3]. As adolescence begins, females show a pronounced rise in internalizing symptoms, most notably depression and anxiety [3, 4]. These internalizing problems become the most prevalent form of psychopathology in adolescence [5], while externalizing problems in males tend to persist but represent a smaller part of the overall burden [5, 6].

The transitional period from childhood to adolescence, defined here as early adolescence spanning ages 9 to 12, is increasingly recognized as a critical period for brain maturation and emotional development [7–10]. From an endocrine perspective, early adolescence involves the onset of two distinct but overlapping maturational processes: gonadarche and adrenarche. Although closely interrelated, these processes are governed by distinct control mechanisms and can occur independently [11–13]. Gonadarche involves activation of the hypothalamic-pituitary-gonadal (HPG) axis, leading to the production of gonadal testosterone and estradiol and the visible physical changes of puberty, including breast development in females, testicular growth in males, pubic and axillary hair growth and accelerated height gain in both sexes [14]. This process is widely considered the hormonal hallmark of puberty and both its timing and progression have been repeatedly linked to mental health outcomes [11, 12, 15–17]. In contrast, adrenarche precedes puberty and marks the endocrine transition from childhood to adolescence [18, 19]. This often underrecognized yet essential phase is characterized by increased secretion of androgens, particularly dehydroepiandrosterone (DHEA) and its sulfated form (DHEA-S), from the adrenal glands [13, 14, 18]. Within in the central nervous system, DHEA and DHEA-S act as negative noncompetitive modulators of GABA_A_ receptors, potentiate glutaminergic N-methyl-D-aspartate (NMDA) receptors, and increase neurite differentiation and neurogenesis, suggesting a role in neurodevelopment [20–22]. Fluctuations in adrenal androgens during early adolescence may influence neural circuits involved in emotion regulation, stress reactivity, and social behavior; processes that undergo substantial refinement during that period of development and are critical for mental health vulnerability [8, 23–25].

Building on this neurobiological rationale for a potential link between endocrine transitions and psychopathological symptoms, several studies have investigated associations between DHEA and youth psychopathology. These studies have been dominated by cross-sectional designs [26–32], with only limited longitudinal investigations [25, 33–35]. Some studies report positive associations between DHEA levels and aggression or conduct problems [26, 28, 29], while DHEA-S levels have also been linked to fewer externalizing symptoms in adolescent males [36]. For internalizing problems, both positive and negative associations between DHEA levels and symptom severity have been reported [25, 26, 29, 30, 32]. Other studies did not detect consistent associations between DHEA or DHEA-S levels and psychopathologies [27, 33, 37].

Overall, the literature on DHEA is conflicting and points to heterogeneous and often sex-specific associations, with effect directions varying by developmental stage, outcome domain, and study design [16, 27, 37–39]. A recent systematic review found that current evidence does not permit firm conclusions regarding associations between androgens and adolescent mental health and identified several methodological weaknesses likely contributing to the mixed findings [37]: First, many studies linking higher androgen levels to mental health risks rely on high-risk or clinical samples, creating potential selection or collider bias. Second, longitudinal designs remain scarce, limiting the ability to draw inference on the direction and causality of reported associations. Third, inappropriate adjustment for confounders was common [37]. Additionally, few studies have explicitly targeted early adolescence [25–28], leaving the role of early-adolescent hormone exposure (particularly adrenarche) underexplored, despite proposals that it represents a sensitive period for neurodevelopment, mental health, and the emergence of sex differences [7–9].

Together, these inconsistencies underscore the need for large-scale, longitudinal, sex-stratified investigations of how early-adolescent hormones may be associated with psychopathology at later stages of puberty. To address these gaps, we analyzed data from the longitudinal, population-based Adolescent Brain Cognitive Development (ABCD) Study [40] to examine whether early-adolescent adrenal androgen levels (DHEA) are associated with externalizing and internalizing psychopathology in later adolescence. By analyzing sex-specific patterns in endocrine markers, we sought to clarify the role of hormones in shaping sex-specific mental health trajectories across adolescence.

## Methods

### Sample description and exclusion criteria

This study analyzed data from the Adolescent Brain Cognitive Development (ABCD) Study, a large longitudinal, population-based cohort comprising 11 868 children and adolescents recruited across 21 sites in the United States [40]. The ABCD Study is a National Institutes of Health (NIH)-supported research project designed to examine brain, cognitive, and behavioral development from late childhood into adolescence [41]. ABCD data are distributed through the National Institute of Mental Health Data Archive (NDA) under controlled access. For this analysis, we used release 5.1 of the dataset (10.15154/z563-zd24).

The ABCD Study conducts annual assessments, including the collection of hormonal saliva samples. The release includes data from baseline (*N* = 11 868, mean age = 9.91 ± 0.62 years, 47.8% female), 1-year (*N* = 11 220, mean age = 10.92 ± 0.64 years, 47.7% female), 2-year (*N* = 10 973, mean age = 12.03 ± 0.67 years, 47.5% female), 3-year (*N* = 10 336, mean age = 12.91 ± 0.65 years, 47.5% female) and 4-year follow-ups (*N* = 4 754, mean age = 14.08 ± 0.68 years, 47.6% female).

Data quality was assessed at two levels (Supplementary Fig. S1). At the subject level, entire participants were excluded if they had missing or implausible key demographic data (missing age, missing race/ethnicity, missing or implausible BMI values), reported congenital differences in sex development, or completely lacked hormonal measurements at both baseline and 1-year follow-up, rendering them unable to contribute to the current study. At the observation level, specific time points were excluded as part of hormone-specific quality control procedures (e.g., refusal or inability to provide saliva sample, sex mismatch, technical assay concerns) and availability of CBCL outcome measures. Additionally, estradiol was assessed only in females per ABCD protocol [42].

These criteria resulted in 45 033 included observations from up to *N* = 11 696 (98.6%) participants (Table 1), where each observation corresponds to a participant’s measurement at a single time point. Models were fitted using this full set of eligible participants, with the number of participants in each model varying depending on the availability of predictor variables. For the 2-year data, DHEA models included up to *N* = 10 210 (male: *N* = 5 327, female: *N* = 4 883); for the 3-year data up to *N* = 9 448 (male: *N* = 4 946, female: *N* = 4 502); and for the 4-year data up to *N* = 4 348 (male: *N* = 2 272, female: *N* = 2 076). Models including ratio values or additional covariates had smaller sample sizes. Across all follow-ups, excluded participants did not differ in age compared to included participants but were more likely to be of black or hispanic backgrounds and more frequently came from lower-income families (Supplementary Tables S1–S3).

**Table 1:** Baseline Characteristics of the Total and Sex-Stratified Sample.

Characteristics	Total Sample (*N* = 11 696)	Males ^a^ (*N* = 6 094)	Females ^a^ (*N* = 5 602)
**Age, mean years (SD)**	9.91 (0.6)	9.93 (0.6)	9.90 (0.6)
**Race/Ethnicity, N (%)**			
White	6 099 (52.1)	3 232 (53.0)	2 867 (51.2)
Black	1 746 (14.9)	872 (14.3)	874 (15.6)
Hispanic	2 369 (20.3)	1 233 (20.2)	1136 (20.3)
Asian	251 (2.1)	121 (2.0)	130 (2.3)
Other	1 231 (10.5)	636 (10.4)	595 (10.6)
**BMI SDS** ^b^**, mean (SD)**	0.43 (1.2)	0.45 (1.2)	0.41 (1.2)
**Weight Category** ^c^**, N (%)**			
Underweight	416 (3.6)	197 (3.2)	219 (3.9)
Normal weight	7 528 (64.4)	3 907 (64.1)	3 621 (64.6)
Overweight	1 766 (15.1)	908 (14.9)	858 (15.3)
Obese	1 986 (17.0)	1 082 (17.8)	904 (16.1)
**PDS Sum Score** ^d^**, mean (SD)**	8.00 (2.4)	7.20 (1.9)	8.87 (2.6)
**PDS Category (Pubertal stage), N (%)** ^e^			
Prepubertal	5 762 (51.3)	4 101 (70.1)	1 661 (30.8)
Early Pubertal	2 665 (23.7)	1 399 (23.9)	1 266 (23.5)
Midpubertal	2 632 (23.4)	314 (5.2)	2 318 (43.0)
Late Pubertal	170 (1.5)	29 (0.5)	141 (2.6)
Post Pubertal	10 (0.1)	<10 ^g^	<10 ^g^
**Physical Activity** ^f^**, (h/week), mean (SD)**	6.1 (6.4)	6.7 (6.8)	5.4 (5.9)

Total sample size refers to all participants who were not excluded based on the criteria outlined in Supplementary Fig. S1. The models drew from this total sample, with the number of participants in each model varying depending on the availability of predictor variables. Maximum sample sizes for DHEA were *N* = 10 210 (male: *N* = 5 327; female: *N* = 4 883) for the 2-year data, *N* = 9 448 (male: *N* = 4 946; female: *N* = 4 502) for the 3-year data, and *N* = 4 348 (male: *N* = 2 272; female: *N* = 2 076) for the 4-year data. Sample sizes were smaller for models including ratio values or additional covariates.

*N* number, *SD* standard deviation, *BMI SDS* body mass index standard deviation score, *PDS* pubertal development scale, *H/Week* hours per week.

^a^Sex assigned at birth as reported by parents.

^b^BMI z-score adjusted for age and sex, based on the 2000 CDC growth reference data for U.S. children and adolescents [82], indicating deviation from the mean BMI of the reference population.

^c^Based on BMI-z-scores: underweight (<−1.645), normal weight (−1.645 to < 1.036), overweight (1.036 to < 1.645), obese (>1.645).

^d^Sum of all item scores on the Pubertal Development Scale (PDS, parent-report [83]) at baseline; higher values indicate more advanced pubertal development.

^e^Categorical pubertal stages (prepubertal, early, mid, late, postpubertal) were taken from the ABCD dataset (sex-specific) for descriptive purposes; the PDS sum score was used in statistical models.

^f^Weekly hours of parent-reported physical activity (e.g., sports, dance, martial arts), calculated as: (days/week × hours/session) per activity, summed across all activities.

^g^Please note that, in accordance with ABCD study privacy protection policies, numbers fewer than 10 are suppressed.

### Ethics

Written informed consent was obtained from legal guardians and assent from all participating children. The ABCD protocol was approved by the central institutional review board at UC San Diego and, at some sites, additionally by local institutional review boards. Data use for this study was approved by the Ethics Committee of the Medical Faculty, University of Duisburg-Essen (24-12040-BO). All methods were performed in accordance with the relevant guidelines and regulations.

### Exposure and outcome

To examine whether early hormonal profiles predict later mental health outcomes, we used hormone levels (averaged across baseline and 1-year follow-up) as well as hormone changes (ratio of 2-year follow-up to baseline) to predict CBCL scores assessed at the 2-, 3-, and 4-year follow-ups.

### Exposure: hormone levels and changes

As part of the ABCD study protocol, salivary levels of DHEA, testosterone, and estradiol (females only) were assayed using the passive drool method and analyzed via Salimetrics enzyme immunoassay kits [42]. At each time point, each hormone sample was assayed in two technical replicates. The final hormone value per time point was calculated as the arithmetic mean of both replicates; if only one replicate was available, that single value was used. As raw hormone concentrations were heavily right skewed, log-transformation of the mean hormone concentrations was performed to approximate a normal distribution (see Supplementary Figs. S2-S3).

Values falling below the lower limit of detection (LLD; DHEA: <5 pg/ml, *N* = 629; testosterone: <1 pg/ml, *N* < 10; estradiol: <0.1 pg/ml, *N* = 225) were imputed on the log-transformed scale using truncated normal regression models. Imputation, rather than exclusion, was applied to retain information on low hormone concentrations. Imputations were conducted separately for each hormone and time point. Sex and age were included as predictors for imputation to retain biological plausibility. Imputed values remained below the respective LLD and approximated a truncated normal distribution consistent with the expected distribution of log-transformed hormone levels (see Supplementary Figs. S6-S11).

The final hormone values were then used to derive early-adolescent hormone levels and changes: To estimate hormonal levels throughout early adolescence and to reduce the influence of short-term fluctuations, we computed the hormone level as the arithmetic mean of the log-transformed baseline (age 9.91 ± 0.62 years) and 1-year follow-up (age 10.92 ± 0.64 years) values per hormone and participant. If only baseline (*N* = 1 809 cases, 16.3%) or 1-year follow-up (*N* = 1 665 cases, 15.0%) was available, that single time point was used. To capture expected changes in hormone concentrations associated with age and pubertal progression, a hormone change variable was calculated as the natural logarithm of the ratio of the 2-year follow-up (age 12.03 ± 0.67 years) value to the baseline value. Our analyses focus on DHEA, which serves as a biomarker of adrenarche and reflects the predominance of adrenal androgens during this developmental phase [8, 13]. In addition, we conducted exploratory analyses including estradiol and testosterone as further pubertal hormones and related sex steroids.

### Outcome: Child Behavior Checklist (CBCL)

Psychopathology was assessed annually using the parent-report Child Behavior Checklist (CBCL), a standardized questionnaire for evaluating emotional and behavioral problems in children and adolescents over the past six months. The 113 items are rated on a three-point Likert scale and are used to compute summary scores for Total, Internalizing, and Externalizing Problems, with higher scores reflecting greater difficulties [43].

To assess whether early hormonal profiles predicted later mental health outcomes, CBCL data from the 2-year, 3-year, and 4-year follow-up assessments served as outcome variables in longitudinal analyses. For primary analyses, we used the raw scores of the syndrome-oriented CBCL scales, namely “CBCL Externalizing” (comprising Rule-Breaking and Aggressive Behavior) and “CBCL Internalizing” (comprising Anxious/Depressed, Withdrawn/Depressed, and Somatic Complaints). CBCL data availability decreased across follow-ups, with nearly complete coverage up to 3 years (DHEA data: 3-year *N* = 9 448, 85.3%). For the 4-year follow-up, only about half of the sample was included in release 5.1, resulting in CBCL availability of *N* = 4 348 (39.3%, Supplementary Fig. S1).

### Covariates

To identify relevant confounders, we used the framework of Directed Acyclic Graphs (DAG, constructed in DAGitty.net [44], Supplementary Fig. S5). This approach allowed us to systematically consider relationships not only between exposure, covariates, and outcome, but also among potential covariates. Based on this framework, the following minimally sufficient adjustment set to adjust for potential confounding was identified: age at baseline (continuous), parent-reported race/ethnicity (five-category factor), BMI standard deviation score at baseline and physical activity at baseline (for rationale of covariate selection, see Supplemental Material and Supplementary Fig. S5).

### Statistical analysis

After evaluating assumptions of linear modeling (see Supplementary Table S5-S7, Supplementary Figs. S12–S14), we estimated separate linear mixed-effects models (LMMs) for each hormone and outcome, including the above-mentioned covariates, a random intercept for study site, and a random intercept for family nested within study site to account for clustering. All hormone and outcome variables were standardized, so that the resulting beta estimates represent changes in the outcome (i.e. CBCL scales) in standard deviations per one standard deviation change in the exposure (i.e. log-transformed DHEA). All models were stratified by sex, given possible differences in hormonal systems between males and females and the study’s focus on sex-specific effects. In addition, we tested for sex-by-DHEA interaction effects to assess whether associations between DHEA levels and mental health outcomes differed by sex across follow-up years and included a DHEA × age interaction to explore potential age-dependent effects. To explore the potential clinical relevance of the results, two further analyses were conducted: First, we estimated adjusted risk ratios (RRs) for the association between early-adolescent hormone levels (averaged across baseline and 1-year follow up) or changes (ratio of 2-year follow-up to baseline values) with CBCL defined thresholds of internalizing and externalizing problems at 2-, 3-, and 4-year follow-ups. Risk ratios were obtained from binomial regression models with a log link, adjusted for the same covariates as the main analyses and stratified by sex. CBCL outcomes were categorized into borderline (T-score ≥ 60) and clinical (T-score ≥ 64) ranges. A detailed description of this analysis is provided in the Supplementary Results (Risk Ratio Analysis). Second, we explored associations between DHEA levels and changes within the six DSM-5-oriented CBCL subscales (Depression, Anxiety, Attention-Deficit/Hyperactivity Disorder (ADHD), Oppositional Defiant Disorder (ODD), Conduct Disorder (CD), and Somatic Problems) [45], which more closely align with clinical diagnostic categories.

### Sensitivity and secondary analyses

To assess the robustness of our main findings, we conducted several sensitivity analyses. For the hormone exposure, we repeated analyses using baseline and 1-year follow-up DHEA values separately rather than their average. Given that CBCL outcome scores represent count data with a right-skewed distribution (Supplementary Fig. S4) and that QQ-plots indicated a violation of the normality assumption of residuals (Supplementary Figs. S12-S13), we re-estimated all models using negative binomial mixed models. In addition, we repeated analyses using Brief Problem Monitor (BPM) Scores as self-report measures instead of parent-report CBCL.

To account for potential confounding, we excluded participants reporting psychotropic or hormone-related medication use at baseline (*N* = 1 241, 10.6%). Additional sensitivity analyses adjusted for pubertal status at baseline and at the 3-year follow-up to account for changes in pubertal development over time as well as adjusting for time interval between baseline and 2-year follow-up in the DHEA change variable. To address potential bias from imputation of hormone values below the lower limit of detection, analyses were repeated excluding participants with such DHEA values at the 3-year follow up (*N* < 10). To examine potentially influential outliers, we further employed winsorized models. Finally, accounting for prior symptomatology (i.e., higher CBCL scores at baseline predicting higher scores at later time points), an expanded model included baseline CBCL scores as an additional covariate.

As secondary analyses, we repeated all models using early-adolescent testosterone and estradiol levels (females only) as alternative exposures and investigated the relationship between hormones (DHEA, testosterone and estradiol).

## Results

We tested whether early-adolescent DHEA levels (averaged across baseline and 1-year follow up) or changes (the ratio of 2-year follow-up to baseline values) predicted later psychopathology by modeling associations with CBCL outcomes across follow-ups. All associations were further examined by testing sex-by-DHEA interaction effects, translating results into risk ratios for developing CBCL scores in the borderline or clinical range for each respective outcome, and examining DSM-5-oriented CBCL subscales. Primary analyses focused on the relationship between DHEA and CBCL Externalizing and Internalizing scores at the 3-year follow-up, as it represented both the latest available assessment with near-complete data coverage in ABCD dataset version 5.1. Further, the investigation was expanded to the 2-year and 4-year follow-up and to include testosterone and estradiol (females only), assessing consistency across hormones and time points.

### The effect of DHEA on psychopathology

Early-adolescent DHEA levels (mean age males 10.41 ± 0.64 years, mean age females 10.38 ± 0.64 years) differed between sexes, with females exhibiting higher salivary DHEA levels than males (males = 57.59 ± 40.49 pg/ml; females = 78.16 ± 55.58 pg/ml; t-test: <0.001).

#### DHEA and CBCL outcomes at 3-year follow-up

**Fig. 1:**
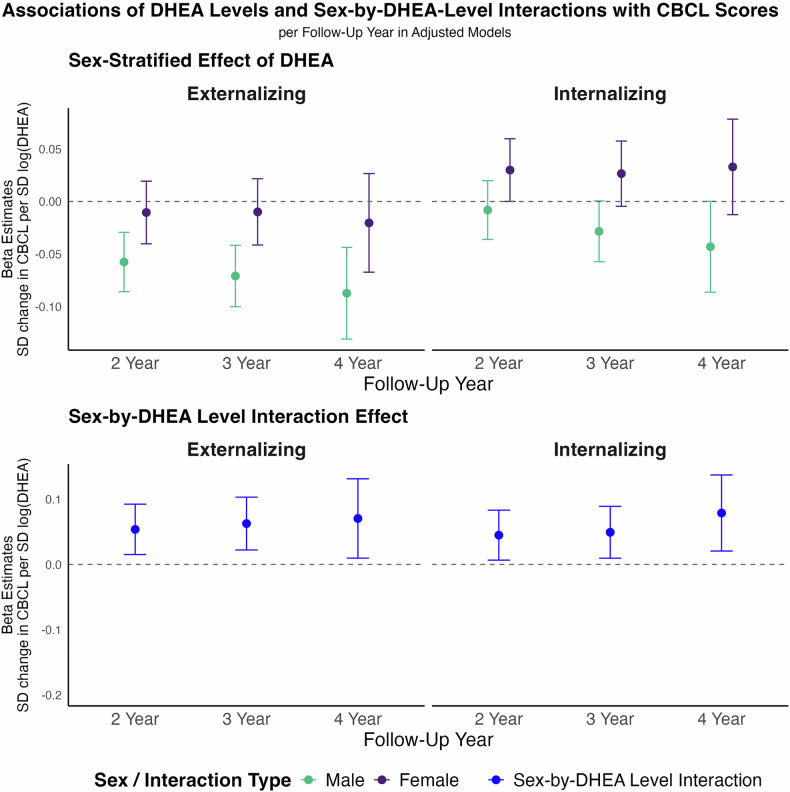
Sex-stratified associations and sex-by-DHEA Level interaction effects of DHEA on Child Behavior Checklist (CBCL) scores across follow-up years, derived from linear mixed models. DHEA levels were defined as the arithmetic mean of the log-transformed baseline and 1-year follow-up DHEA values per participant. Panels display standardized beta estimates for the association between DHEA levels and CBCL outcomes (Externalizing and Internalizing scores) at 2-, 3-, and 4-year follow-up. Top row: Sex-stratified effects of DHEA for males (teal) and females (purple). Bottom row: Sex-by-DHEA interaction effects (blue) indicate the differential association according to sex. The y-axis represents standardized beta estimates (standard deviation change in CBCL score per standard deviation change in log-transformed DHEA level); error bars denote 95% CIs. Models are adjusted for age, race/ethnicity, BMI SDS, and physical activity, all assessed at baseline. Sample sizes for both Externalizing and Internalizing scores are at the 2-year follow-up: *N* = 5 327 males and *N* = 4 883 females; 3-year follow-up: *N* = 4 946 males and *N* = 4 502 females; 4-year follow-up: *N* = 2 272 males and *N* = 2 076 females, respectively. 95%-CI 95% confidence interval, BMI SDS body mass index standard deviation score, CBCL child behavior checklist, DHEA dehydroepiandrosterone, N number, SD standard deviation.

At 3-year follow up (mean age 12.91 ± 0.65 years) higher DHEA levels in males were associated with lower externalizing symptoms scores (Fig. 1). This association remained after adjusting for covariates, showing an estimated effect size of β = –0.07 SD change of CBCL per SD-change of log-transformed DHEA levels (95% CI [–0.10, –0.04]). The association with internalizing symptoms in males was weaker, with an estimated effect size of ß = −0.03, 95% CI [−0.06, 0.00]. In females, DHEA levels were neither associated with externalizing (β = −0.01, 95% CI [–0.04, 0.02]) nor with internalizing symptoms (β = 0.03, 95% CI [0.00, 0.06]). Changes in DHEA throughout early adolescence, calculated as the 2-year follow-up to baseline ratio, did not predict CBCL outcomes in either sex (adjusted models: males – externalizing: β = 0.01, 95% CI [–0.02, 0.05]; internalizing: β = 0.00, 95% CI [–0.04, 0.04]; females – externalizing: β = 0.00, 95% CI [–0.03, 0.04]; internalizing: β = –0.02, 95% CI [–0.06, 0.02]).

#### Longitudinal stability of DHEA effects

To test the longitudinal stability of our findings and investigate potential developmental shifts, we extended our analyses to 2- and 4-year follow-ups (Fig. 1). The negative association between DHEA levels and later externalizing symptoms in males increased from β = –0.06 (95% CI [–0.09, –0.03]) at 2-year follow-up to β = –0.09 (95% CI [–0.13, –0.04]) at 4-year follow-up, though the magnitude of the effect remained small. Internalizing symptoms in males also showed negative associations at later follow-ups (2-year follow-up: β = –0.01, 95% CI [–0.04, 0.02]; 4-year follow-up: β = –0.04, 95% CI [–0.09, 0.00]). In females, associations for both externalizing and internalizing symptoms were minimal and fluctuated around zero across all follow-ups.

#### Sex-by-DHEA interaction effects

For DHEA level, interaction estimates for both externalizing and internalizing symptoms showed small but increasing sex × DHEA level interaction effects over time (Fig. 1): For externalizing symptoms, interaction estimates increased from β = 0.05 (95% CI [0.02, 0.09]) at 2-year follow-up to β = 0.07 (95% CI [0.01, 0.13]) at 4-year follow-up. A similar pattern was observed for internalizing symptoms (2-year follow-up: β = 0.04, 95% CI [0.00, 0.08]; 4-year follow-up: β = 0.08, 95% CI [0.02, 0.14]). All sex × DHEA change interaction terms remained non-significant (Supplementary Table S27). To formally assess whether associations varied with age, we conducted sensitivity analyses including DHEA × age interaction terms. These interactions were not statistically significant for either CBCL domain or sex (Supplementary Table S28).

#### Risk for symptoms reaching clinical thresholds

**Fig. 2:**
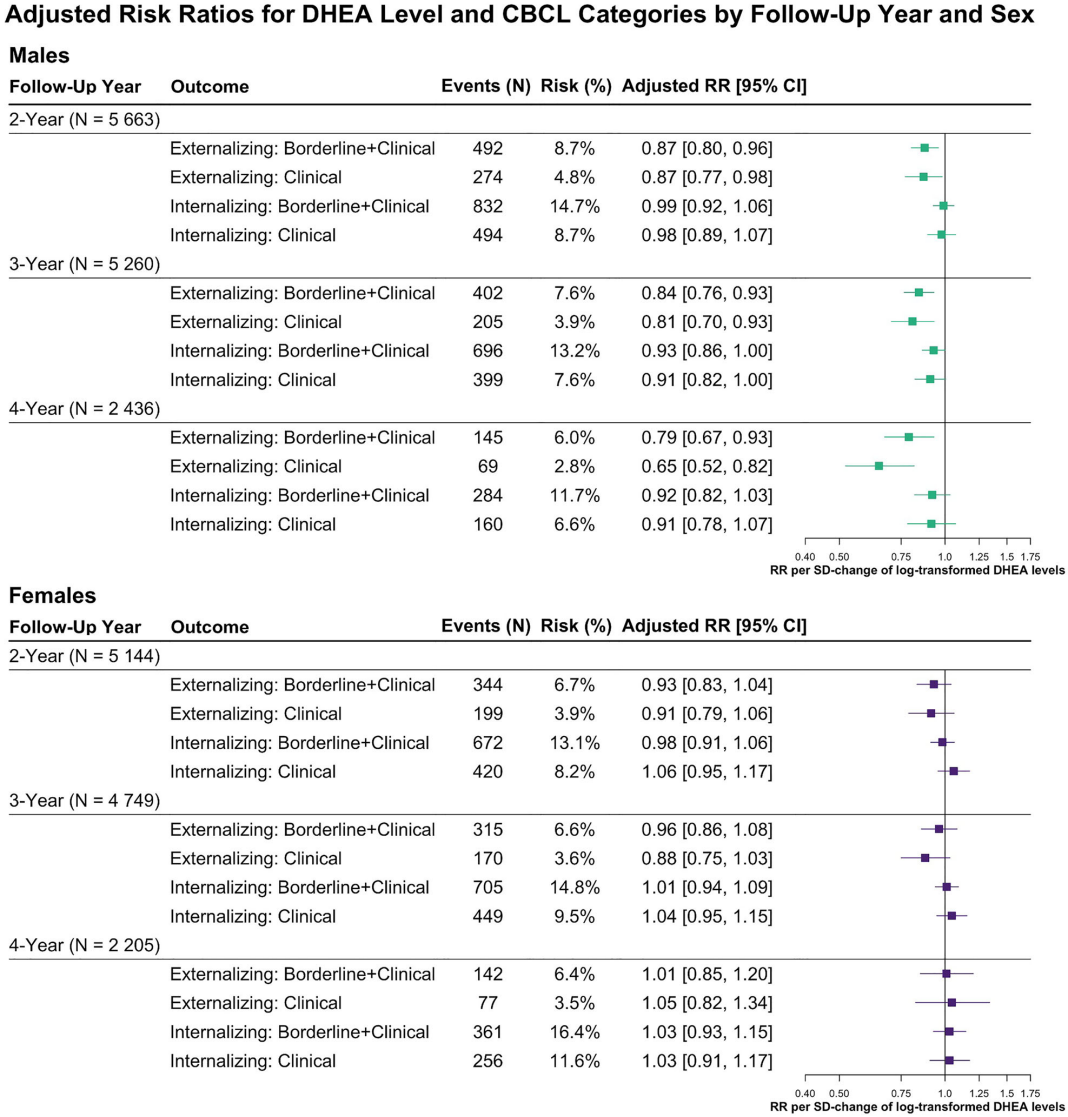
Sex-stratified risk ratios for the associations between DHEA levels and Child Behavior Checklist (CBCL) diagnostic categories (Externalizing and Internalizing, each divided into Borderline+Clinical and Clinical) across 2-, 3-, and 4-year follow-ups. Adjusted risk ratios (RRs) were estimated using binomial regression models. DHEA levels were defined as the arithmetic mean of the log-transformed baseline and 1-year follow-up DHEA values per participant. Panels present adjusted RRs and 95% CIs for the associations between DHEA levels and CBCL outcomes separately for males (top panel, teal) and females (bottom panel, purple). The y-axis lists CBCL outcome categories; the x-axis shows adjusted RRs per SD-change of log-transformed DHEA levels. Squares represent point estimates; horizontal bars indicate 95% CIs. CBCL Score Interpretation: Normal Range: T-score ≤ 59; Borderline + Clinical Range: T-score ≥ 60; Clinical Range: T-score ≥ 64. Events (N) indicates the number of participants meeting the CBCL threshold at the respective follow-up. Risk (%) represents the proportion of participants in that category relative to the total number with available data. Models are adjusted for age, race/ethnicity, BMI SDS, and physical activity, all assessed at baseline. Sample sizes for both Externalizing and Internalizing scores are at the 2-year follow-up: *N* = 5 663 males and *N* = 5 144 females; 3-year follow-up: *N* = 5 260 males and *N* = 4 749 females; 4-year follow-up: *N* = 2 436 males and *N* = 2 205 females, respectively. 95% CI 95% confidence interval, BMI SDS body mass index standard deviation score, CBCL child behavior checklist, DHEA dehydroepiandrosterone, N number, RR risk ratio, SD standard deviation.

The effects of DHEA levels on CBCL scores translated into differences in the risk of exceeding established thresholds for borderline (T-score ≥ 60) and clinical (T-score ≥ 64) levels of psychopathological symptoms (Fig. 2). In males, higher DHEA levels were linked to a reduced risk to reach borderline or clinical-levels of externalizing symptom severity (T-score ≥ 60) across all follow-up years: The adjusted risk ratio per SD-change of log-transformed DHEA levels was 0.87 (95% CI [0.80, 0.96]) at 2-year follow-up and further declined to 0.79 (95% CI [0.67, 0.93]) at 4-year follow-up. The strongest effect emerged at the clinical cutoff (T ≥ 64) at 4-year follow-up: RR = 0.65 (95% CI [0.52, 0.82]). There was no clear evidence for a relationship between DHEA levels and internalizing problems in males, nor for either symptom domain in females.

#### DHEA and CBCL subdomains

Associations between DHEA levels and DSM-5-oriented subscales were primarily evident in males (Supplementary Figs. S16-18). Higher DHEA levels were linked to lower symptoms of Conduct Problems, Oppositional Defiant Problems, and ADHD across all follow-up years. In females, there was no consistent pattern across subscales, though a transient association with lower ADHD symptoms was observed at the 2-year follow-up. For internalizing symptom subscales, negative associations between DHEA levels and Anxiety and Depression Problems were observed in males at the 3- and 4-year follow-ups, although effect sizes were smaller than those for externalizing symptoms.

### Effects of testosterone and estradiol on psychopathology

**Fig. 3:**
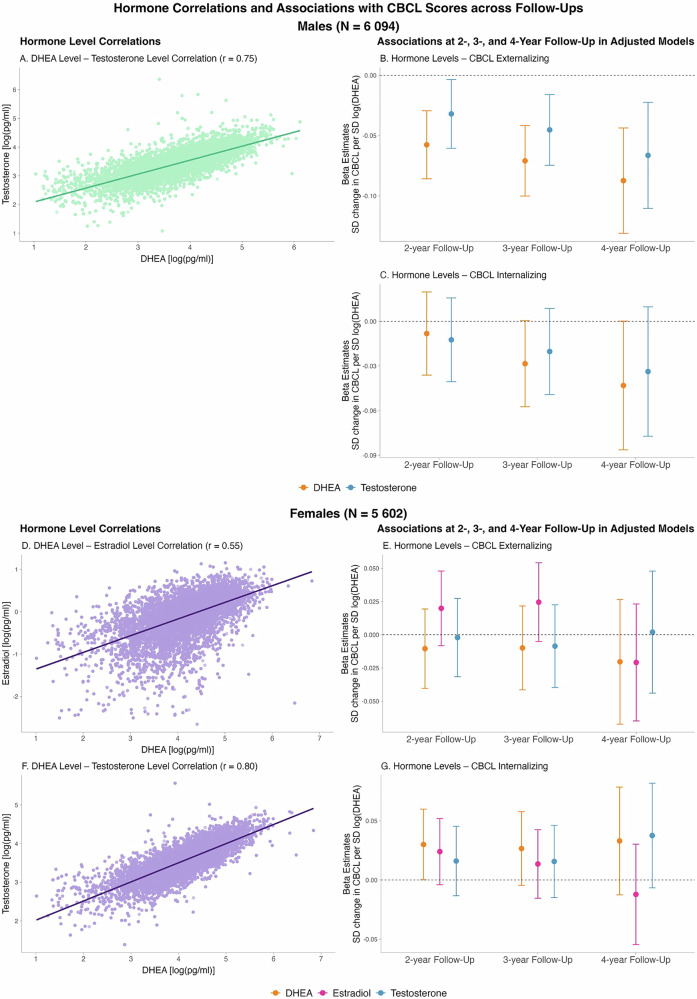
Sex-stratified hormone level correlations and associations between hormone levels and CBCL scores across 2-, 3-, and 4-year follow-ups. **A**–**C** show results for males (*N* = 6 094): (**A**): Pairwise correlation between DHEA and Testosterone with a linear regression line and Pearson’s r. **B,** **C**: Beta estimates (points) and 95% Cis (error bars) for the associations between hormone levels (DHEA, Testosterone) and CBCL Externalizing (**B**) and Internalizing (**C**) scores at 2-, 3-, and 4-year follow-up. Colors: orange: DHEA; pink: Estradiol; blue: Testosterone. **D**–**G** show results for females (*N* = 5 602): (**D,** **F**): Pairwise correlations between DHEA and Estradiol (**D**) and DHEA and Testosterone (**F**) with linear regression lines and Pearson’s r. **E,** **G**: Beta estimates (points) and 95% CIs (error bars) for the associations between hormone levels (DHEA, Estradiol, Testosterone) and CBCL Externalizing (**E**) and Internalizing (**G**) scores at 2-, 3-, and 4-year follow-up. Beta estimates reflect SD change of CBCL per SD-change of log-transformed hormone level. DHEA is re-shown from Fig. 1 for comparison to the effects of testosterone and estradiol. Hormone levels were defined as the arithmetic mean of the log-transformed baseline and 1-year follow-up hormone values per participant [log(pg/ml)]. All models are adjusted for age, race/ethnicity, BMI SDS and physical activity, all assessed at baseline. 95%-CI 95% confidence interval, BMI SDS body mass index standard deviation score, CBCL child behavior checklist, DHEA dehydroepiandrosterone, r Pearson’s R correlation coefficient, SD standard deviation.

Analyses revealed strong positive correlations between DHEA levels and both testosterone (males: mean r = 0.75, females: mean r = 0.80) and estradiol levels (females only, mean r = 0.55, see Fig. 3 and Supplementary Table S26 across all time points and remained high after controlling for pubertal status. Strong correlations indicate that multicollinearity prevents statistically disentangling the independent effects of DHEA, testosterone, and estradiol.

In males, effects of early-adolescent testosterone levels (averaged across baseline and 1-year follow-up) on CBCL domains showed a similar pattern to the effects of DHEA levels: Testosterone levels showed consistent negative associations with externalizing symptoms and smaller effect estimates with internalizing symptoms across all time points (Fig. 3). Confidence intervals were generally wider for testosterone than for DHEA. In females, none of the three hormones showed consistent associations with CBCL outcomes. Effect estimates varied across time points, and all confidence intervals included zero (Fig. 3).

To test whether testosterone provided additional explanatory power beyond adrenal DHEA, we included testosterone as an additional exposure in the adjusted 3-year mixed-effects models. Adding testosterone did not meaningfully increase the proportion of variance explained in CBCL externalizing or internalizing scores in either males or females (see Supplemental Results).

### Sensitivity analyses

To test the robustness of our findings, we conducted several sensitivity analyses addressing methodological and biological concerns. Re-estimating models using baseline and 1-year follow-up DHEA values separately rather than their average (Supplementary Tables S20-S21), using negative binomial regression to account for the skewed distribution of CBCL scores (Supplementary Tables S8–S9), and employing Brief Problem Monitor (BPM) Scores as self-report measures instead of parent-report CBCL (Supplementary Tables S18-S19) yielded results similar to the primary findings.

Similarly, excluding participants taking psychotropic or hormone-related medication at baseline (Supplementary Tables S10-S11), adjusting for pubertal status at baseline (Supplementary Tables S12-S13) and at 3-year follow-up (Supplementary Tables S14-S15), and adjusting for time interval between baseline and 2-year follow-up in the DHEA change variable (Supplementary Tables S22-S23) did not materially alter the results. Results were also consistent when excluding hormone values below the detection threshold rather than imputing them (Supplementary Tables S16-S17) and when assessing the influence of extreme values using winsorized models (Supplementary Tables S24-S25). Controlling for baseline CBCL scores slightly attenuated the effect estimates of DHEA levels on externalizing CBCL scores in males, although the associations remained significant. The sex-by-DHEA interaction estimates remained largely unchanged when including baseline CBCL scores as a covariate in the model (see Supplementary Fig. S15 and Supplementary Table S27).

## Discussion

This longitudinal study investigated whether DHEA levels in early adolescence predicted later externalizing and internalizing symptoms in a large population-based cohort of youth (up to *N* = 11 696). To capture hormone levels throughout early adolescence (age 9–12 years) and reduce the influence of short-term fluctuations, hormone concentrations were averaged between log-transformed baseline and 1-year values. Outcomes were assessed up to four years later (mean age males 14.09 ± 0.69 years, mean age females 14.07 ± 0.67 years).

### Early-adolescent DHEA-levels are linked to lower externalizing and internalizing symptoms in males

Our findings indicate that higher early-adolescent DHEA levels are associated with lower externalizing symptom severity in males. This association was consistent across all externalizing symptom subscales (Conduct, Oppositional Defiant, and ADHD Problems) and across all follow-ups. Overall, effect sizes were modest but robust, translating into a meaningful reduction in the likelihood of developing borderline or clinical-level externalizing symptoms. Similar, though less consistent, effects were observed for internalizing problems.

In contrast, studies in clinical samples have reported associations between higher adrenal androgen levels and externalizing symptoms in adolescents with conduct disorder [28, 46, 47]. Such associations have also been discussed in a systematic review concluding that adrenal androgens may serve as risk markers for psychopathology [8]. However, findings from clinical samples may not necessarily generalize to population-based cohorts, as these samples often represent more severe or selected phenotypes and may be prone to selection bias [37]. While clinical or high-risk samples are well suited to examine within-disorder correlates or severe phenotypes, their findings may not readily generalize to population-level or etiological associations, highlighting the value of population-based designs such as the present study.

Existing population-based studies have yielded mixed results [25–27, 29–31, 37]. One cross-sectional study found a negative association between DHEA-S and self-reported externalizing symptoms in 226 boys aged 11–12 years [36], whereas a longitudinal study of 56 boys aged 10–14 years reported positive associations between DHEA/DHEA-S and symptoms of depression and anxiety [30]. Others found no such associations [27, 37]. The heterogeneity in findings likely reflects methodological variation, as outlined by a recent systematic review [37], particularly in sample composition, hormonal assessment, and covariate selection. In this study, we leveraged population-based data, averaged hormone concentrations over time to reduce short-term fluctuations in hormone values, and examined the longitudinal influence of DHEA on mental health outcomes while adjusting for age, race/ethnicity, BMI, and physical activity. These design choices not only address methodological limitations of prior research [37], such as reliance on high-risk or clinical samples, scarcity of longitudinal designs, and insufficient confounder control, but also extend the field conceptually by focusing on early-adolescent hormone levels as predictors of later psychopathology.

Our findings support models proposing that the transition from childhood to adolescence, endocrinologically marked by adrenarche, represents a sensitive window for neurodevelopment [7–9]. Although gonadarche has often been emphasized as a key pubertal milestone [14], our results suggest that adrenarche, an earlier endocrine transition, may also play a role in shaping mental health trajectories. DHEA, a hormonal surrogate marker of adrenarche [13, 18], may influence behavior via central neurophysiological pathways. As a neurosteroid, DHEA modulates GABAergic and glutamatergic signaling, alters cortical excitability, and shapes neurodevelopment by promoting neurite outgrowth and neurogenesis [18, 20, 22]. Adrenarche-related hormonal shifts are thought to influence frontolimbic brain systems involved in impulse control and executive function [48, 49], which are closely related to externalizing psychopathology [50, 51], including regions such as the prefrontal cortex and amygdala [25, 52, 53]. Future studies should clarify the neuropathophysiological mechanisms underlying the current findings and specifically address the sex-specific role of adrenal androgens for frontolimbic neurodevelopment in early-adolescent males.

### Early-adolescent DHEA-levels have sex-specific effects on adolescent mental health

Adolescence marks a notable shift in the prevalence of mental health problems between sexes, with boys showing more externalizing symptoms during childhood and internalizing symptoms increasing sharply during adolescence in females [3, 4], becoming the most prevalent form of psychopathology in adolescence overall [5].

In our study, higher DHEA levels were consistently associated with lower externalizing symptoms in boys, while no consistent associations were observed in girls. These sex-specific findings were longitudinally stable across 2- to 4-year follow-ups and descriptively even showed a slight increase in magnitude for externalizing symptoms in boys, indicating persistent sex-specific associations of DHEA with adolescent mental health. Although sex × DHEA interaction estimates were descriptively larger at later follow-ups, formal DHEA × age tests were not significant.

Our findings contribute to a body of research suggesting that associations between adrenal androgens and mental health outcomes may be moderated by sex. Consistent with our findings, several studies reported null associations in girls [27, 33, 54, 55] and some reported stronger associations in boys compared to girls [29–31]. The lack of associations in girls is consistent with previous findings [27, 33, 37] and is notable given the link between earlier pubertal timing and adverse mental health outcomes such as depression in females [56, 57]. These findings suggest distinct contributions of adrenarche and gonadarche and underscore the importance of examining both developmental periods independently in relation to mental health. It has further been proposed that sex differences may not emerge until later in adolescence [27], a hypothesis broadly consistent with the descriptive trend of increasing sex x DHEA interaction estimates at later follow-up years observed in our analyses.

Our findings further align with the notion that boys and girls differ in their neuroendocrine sensitivity and developmental trajectories. Early adolescence, or juvenile transition, is proposed as a neurodevelopmental and psychosocial sensitive period for the development of sex differences in neuroendocrine regulation and behavior [7]. During this period, sex differences in aggression [58, 59] and attachment patterns [60] become more apparent. Furthermore, genetic and environmental contributions to behavior vary by both age and sex, becoming pronounced in middle childhood, with stronger genetic effects in boys [61, 62]. Those neurodevelopmental changes are thought to be, at least in part, mediated by adrenarche [7], which initiates hormonal cascades promoting sexual differentiation in the brain prior to gonadal puberty [63, 64]. More recent evidence links adrenal androgens such as DHEA to cortical maturation and spontaneous neural activity in early adolescents, indicating that adrenarche actively shapes neurodevelopmental pathways relevant to emotion and cognition [52, 65]. Additionally, interactive effects among adrenal androgens (DHEA, testosterone) and further with stress hormones (e.g., cortisol) have been reported to change during adrenarche and adolescence, impacting brain and behavioral development and contributing to sex-divergent trajectories [52, 66, 67].

Taken together, these findings strengthen the view that early adolescence constitutes a sensitive window with high adaptive plasticity [7, 8], during which adrenal androgens may mediate neurodevelopmental divergence between boys and girls. Extended follow-up studies are needed to assess whether the sex-specific DHEA-psychopathology associations observed here persist into later adolescence and contribute to sex differences in adult mental health.

### Effects of testosterone and estradiol may reflect shared adrenal origin

In our study, testosterone mirrored DHEA effects and showed comparable associations with externalizing and internalizing symptoms in males (Fig. 3), remaining consistent when adjusting for pubertal status. DHEA and its sulfate ester (DHEA-S) are produced by the adrenal glands and are considered hallmark biomarkers of adrenal androgen production and adrenarche [18]. In contrast, estradiol and testosterone are typically viewed as gonadal pubertal hormones, whose production increases during gonadarche via activation of the hypothalamic–pituitary–gonadal axis [14]. However, DHEA and DHEA-S also serve as precursors for more potent sex steroids, including testosterone and estradiol, through peripheral (including gonadal) enzymatic conversion, making them part of a complex and interconnected steroid hormone network [18, 23, 68, 69]. Because adrenarche precedes gonadarche, adrenal-derived pathways likely provide the main source of circulating testosterone and estradiol in late childhood and the early adolescence [23, 68, 69]. Gonadal contributions to circulating testosterone and estradiol are expected to be minimal during early adolescence [18], consistent with evidence that gonadal steroids increase later than adrenal ones [70]. Consistent with a presumably shared adrenal origin, we observed high correlations between DHEA levels and testosterone/estradiol, even after adjusting for current pubertal stage (Supplementary Table S26). The effects of early-adolescent testosterone and estradiol on later CBCL externalizing and internalizing scores largely mirrored those of early-adolescent DHEA levels, and adding testosterone alongside DHEA did not meaningfully increase the variance explained in either outcome (see Supplementary Results). Although we cannot definitively determine the source of circulating testosterone and estradiol in our sample, the findings suggest their associations with psychopathology in early adolescence may reflect shared adrenal activity rather than independent gonadal effects.

Recent studies on the contribution of steroids to adolescent mental health emphasize the importance of viewing steroid hormones as interdependent components within a shared metabolic architecture [71, 72]. Examining hormonal constellations has been proposed as a more robust predictor of mental health outcomes than single hormone values and allows the identification of specific hormonal profiles for psychopathologies [71, 72]. However, such profiling relies on ressource-intensive techniques such as gas or liquid chromatography–mass spectrometry (GC-/LC-MS) [73]. Although GC/LC-MS has been used to link adrenal dysregulation with depression in a clinical sample of adolescents [71, 74], population-based studies employing these methods are still needed. Future studies should use GC/LC-MS-based steroid profiling to simultaneously capture related steroid levels and disentangle adrenal and gonadal contributions to mental health effects in population-based samples.

### Limitations and future directions

Our study has several limitations. First, our findings relied on parent-reported symptom scores from the CBCL. Although widely used to assess psychopathology symptom burden in children and adolescents, the CBCL has recently been subject to critical debate [75]. Originally designed as a screening tool to detect clinical levels of psychopathology in individuals, it was not intended to measure latent psychopathology traits in population-based samples, and its proposed dimensional structure failed to replicate in the ABCD cohort [75]. Recent attempts to develop an improved factor structure have also been unsuccessful [75–77], and no similarly comprehensive alternative measures are available in the ABCD dataset. Given that the limitations of the CBCL are primarily expected to attenuate associations between biological variables and psychopathology [75] (i.e., increase the likelihood of false negatives), we do not expect these limitations to compromise the validity of our current findings.

Second, we were not able to account for all contextual factors that may influence the relationship between adrenal androgens and mental health. Several studies have reported such interactions, for example, modulation of the association between DHEA and internalizing symptoms by salivary alpha-amylase concentrations [36] or effects dependent on the DHEA response to stressful situations [78].

Third, hormone levels were measured via salivary samples using immunoassay (ELISA) methods, which, although widely applied in developmental research, have lower specificity than mass spectrometry–based approaches as GC/LC-MS. Future studies may employ these techniques which also allow to disentangle adrenal and gonadal hormone sources, identify pathway-specific markers, and better understand the role of single hormones on brain development [71, 72].

## Conclusions

Our findings add to a growing, yet heterogeneous, body of research on hormonal influences during the transition from late childhood to early adolescence. Small, but consistently found negative associations between early-adolescent DHEA levels and externalizing symptoms in males suggest a potential protective role of adrenal androgens during early adolescence and were translated to a reduced risk to reach borderline or clinical-levels of symptom severity.

The sex-specific associations, together with their longitudinal consistency, point to a potential role of adrenal androgens in the sex-specific developmental prevalence-shift of mental health problems across puberty and highlight adrenarche as a neurodevelopmental window influencing the emergence of psychiatric symptoms in adolescence.

## Supplementary information

Supplemental Material

## Data Availability

This study used data from the Adolescent Brain Cognitive Development (ABCD) Study, a National Institutes of Health (NIH) research project [79]. ABCD data are available via the National Institute of Mental Health Data Archive (NDA) under controlled access. Researchers can request data access via the NDA data application process (https://nda.nih.gov/abcd). Data access is granted after approval to ensure compliance with ethical guidelines and participant confidentiality. For further information, please visit the official ABCD Study [41] or NDA websites [80].

## Code availability

All statistical analyses were conducted with R version 4.4.3 [81]. The statistical code is available upon reasonable request from the authors.
